# Comparison of online quizzes and standard summative examination for the evaluation and guidance of students in medical bacteriology and mycology

**DOI:** 10.1186/s12909-025-06926-0

**Published:** 2025-03-05

**Authors:** Korakrit Imwattana, Popchai Ngamskulrungroj

**Affiliations:** Department of Microbiology, Faculty of Medicine Siriraj Hospital, Mahidol University, Bangkok, Thailand

**Keywords:** Cognitive domain, Online quiz, Assessment, Medical bacteriology, Medical microbiology

## Abstract

**Background:**

This study aimed to determine the effectiveness of loosely regulated online quizzes in evaluating students’ knowledge outcomes compared to a traditional summative examination, using Medical Bacteriology and Mycology as a model.

**Methods:**

A retrospective review of the student’s academic performances was conducted (*n = *320), analyzing the quiz score as well as how each student did the quizzes. Each parameter was mapped back to the students’ actual summative score to evaluate the correlation.

**Results:**

There was a strong correlation between the online quiz and the summative scores (*p* = 0.013). An in-depth analysis also shows that students who performed well in the summative examination had the following characteristics; [1] they tried to pass the online quizzes in the early attempts (*p < *0.001), [2] continued to revisit the quizzes after passing (*p* = 0.011) and [3] tried further to improve their quiz scores (*p* = 0.002).

**Conclusion:**

Although online quizzes cannot yet replace traditional examinations, they can be an effective tool to assess student’s competence, and careful monitoring of the quiz results can help identify students who may need extra attention. The results also suggest that diligence and intelligence are both important in Medical Bacteriology and Mycology.

## Background

In the past decade, online quiz platforms have become more accessible, ranging from basic platforms to sophisticated tools with increased versatility. Some platform also allows immediate detailed feedback, which further facilitates learning [1, 2], in line with the competency-based framework of medical education, defined as an education framework that focuses on a student’s ability to demonstrate specific competencies rather than the duration that a student takes to complete the course [3, 4]. With such quality, online quizzes have become a potential tool for both teaching and assessment of knowledge competence [5]. Although there might be limitations to the use of online quizzes as an assessment tool, the misuse of such tools may hinder the learning process [6].

The Faculty of Medicine Siriraj Hospital, Mahidol University, Thailand has implemented online quizzes as a part of the assessment scheme in the updated 2021 curriculum. With the new assessment method, students must take and pass online quizzes mapped to each course learning outcome (CLO). Also, students need to pass a summative examination at the end of the course to pass the course. The retention of the summative examination was mainly due to concerns regarding the regulation of online quizzes and their validity as an assessment tool [7]. While this seems to be an effective approach, it contradicts the principle of programmatic assessment which is the key component of a competency-based curriculum [3, 8].

For the curriculum to be fully aligned with the competency-based framework, summative examinations should be replaced by multiple low-stake assessments that students can take at their own pace [8]. The current CLO-based online quizzes are a promising tool with several striking properties; students can take each quiz at their own pace with immediate feedback for further self-improvement. If the concern regarding its validity can be mitigated, it should be an effective tool and a foundation for a competency-based knowledge assessment scheme.

This study aims to identify the parameters obtained from online quiz platforms that are associated with good summative outcomes, and see if they can be used to predict students’ performance, using the Medical Bacteriology and Mycology course – the course mainly focused on basic recall and comprehension knowledge – as a model.

## Methods

### Study population

This study was a retrospective review of the Medical Bacteriology and Mycology class in the 2022 Academic Year at the Faculty of Medicine Siriraj Hospital, Mahidol University, Thailand. There were 320 medical students in each class, all of whom were included in this study.

### Assessment structure

Based on the 2021 Doctor of Medicine curriculum at the Faculty of Medicine Siriraj Hospital, students’ knowledge outcomes must be evaluated both during and at the end of the course. The first part of the evaluation (milestone assessment) could be designed as the teachers saw fit, while the evaluation at the end of the course was fixed to be a single-best answer multiple choice question (SBA-MCQ) examination. In addition, the curriculum implemented a non-compensatory approach to assessment. Each course must assign specific learning outcomes and evaluate them individually.

The milestone assessment of the Medical Bacteriology and Mycology course was designed to incorporate elements of programmatic assessment [8]. A large pool of SBA-MCQ exam items was generated and deposited in the Faculty’s online question bank, which was based on Moodle backbone. Several quizzes were generated corresponding with specific CLOs. During the two months of the course, the students could freely choose to complete each open-book quiz at any time and any place. They could also repeat the quiz as many times as they want. In each attempt, the quiz would be regenerated from the pool of exam items, with immediate short feedback after completion. Students were required to pass each quiz once, with a passing score of 80% or higher, to pass the course. There were a total of 3 online quizzes; two for Medical Bacteriology and one for Medical Mycology.

The summative examination took place approximately one week after course completion. It was a tightly regulated, on-site, closed book SBA-MCQ examination. Briefly, students needed to arrive at the examination venue 15 min before the examination. All personal belongings were not allowed in the examination room. The room was also monitored by academic personnel and security cameras throughout the examination. Students were required to pass the summative examination, with a passing score of 50% or higher; an absolute standard based on the modified Angoff’s method (i.e. a panel of subject matter experts evaluated all test items and defined the minimally competent level) [9], to pass the course.

### Data collection and parameters identification

All the data on the pre-summative quizzes was extracted retrospectively from the database. From the raw data, 7 parameters in Table 1 were defined and used in the subsequent analyses. Each parameter was designed to encapsulate two preferable abstract qualities of medical students: intelligence and diligence. According to the Oxford English Dictionary, intelligence is defined as “the faculty of understanding; intellect,” and diligence is defined as “constant and earnest effort to accomplish what is undertaken; persistent application and endeavor.” In this study, intelligence refers to parameters that mainly assess a student’s baseline knowledge and ability and diligence refers to parameters that mainly assess a student’s effort to improve their competency. This classification aimed to determine whether a student’s final competency level is more related to their inherent knowledge or their effort throughout the course.

**Table 1:** Parameters used in the analyses

		Representing qualities	
Name	Definition	**Intelligence**	**Diligence**
Attempts to pass	The number of attempts the student took to achieve a passing score (80% or higher)	✓	
Time to first pass	The number of days after the beginning of the course the student took to achieve a passing score (80% or higher)	✓	
First passing score	The first score that is 80% or higher	✓	
Total tries	The total number of attempts the student took		✓
Pass/total tries	The ratio of the number of attempts with a passing score (80% or higher) and the total number of attempt	✓	✓
Attempts after the first pass	1 = The student attempted the quiz after receiving a passing score 0 = The student did not attempt the quiz after receiving a passing score		✓
Improvement after the first pass	1 = The student had an improvement after receiving the first passing score 0 = The student did not have an improvement after receiving the first passing score		✓

### Adjustments in the following year

As the correlation between the online Mycology quiz and the summative results was poor in the Academic Year 2022 (see results below), adjustments were made in the following year. The Mycology quiz was available on the same platform, but the students had to come and take the quiz at the designated area with the full regulations stated above (closed book with tester registration). Students could still come and take the quiz as many times as they desired and they need to receive the passing score at least once. All data was collected and analyzed in the same manner.

### Statistical analysis

All statistical analyses were performed using online tools available at Statistics Kingdom [10]. Multiple linear regression with backward stepwise selection was performed to assess the association between the pre-summative parameters in Table 1 with the summative score from the same CLO topic [1]. For the regression analysis, statistical significance was achieved at *p < *0.05. The student’s estimated summative score was then calculated using the model obtained from the regression analysis. The presumptive grade (i.e. pass or not pass) was then determined using this estimated score on the standard summative criterion (pass at 50% or higher). This presumptive grade was then compared to the actual summative grade the student received and a Cohen’s kappa was calculated to assess the correlation.

## Results

### Students’ test-taking pattern

**Fig. 1:**
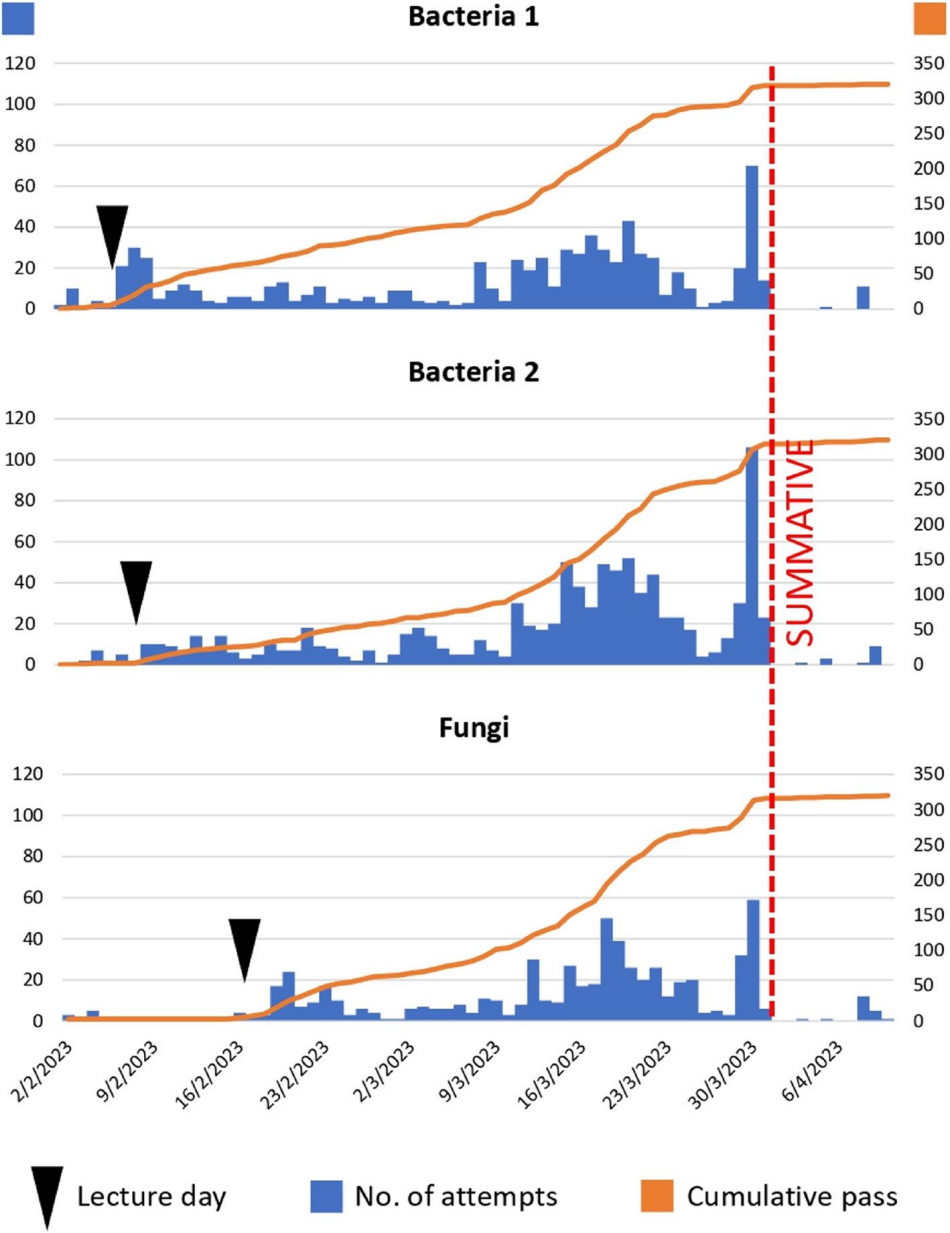
Number of test attempts and the cumulative pass per day for the three online quizzes. The left vertical axis shows the number of test attempts each day while the right vertical axis shows the total number of students with a pass result up until that day (approaching 320 students at the end of the course)

Throughout the three quizzes, the students demonstrated a similar test-taking pattern, as shown in Fig. 1. At the beginning of the course, a few – presumably academically adept – students attempted and passed the quizzes, even before attending the lecture. The number of test attempts spiked a few days after the lecture and gradually decreased. The number of attempts increased again around 2 weeks, and peaked just a few days, before the summative examination. Only a few students did not pass the quizzes at this time and had to re-attempt after the summative examination.

### Predictive parameters

The predictive parameters for the three quizzes in the Academic Year 2022 are summarized in Table 2. Overall, the duration taken before receiving the first passing score (either the number of attempts or the time) was negatively correlated with the summative score, i.e. students who received the passing score sooner tended to receive a higher summative score. Also, students who attempted the quizzes after passing and showed improvement in their scores tended to receive a higher summative score. Interestingly, the first passing score or the total number of attempts or passing attempts were not correlated with the summative score in any analyses.

**Table 2:** Predictive parameters from the regression model

Parameters	Bacteriology quiz 1	Bacteriology quiz 2	Mycology quiz
Attempts to pass	- 2.3 (*p* = 0.0011)	-	-
Time to first pass	- 0.2 (*p* = 0.0014)	- 0.4 (*p < *0.0001)	- 0.3 (*p < *0.0001)
First passing score	-	-	-
Total tries	-	-	-
Pass/total tries	-	-	-
Attempts after the first pass	-	-	10.3 (*p* = 0.0002)
Improvement after the first pass	15.7 (*p < *0.0001)	12.4 (*p* = 0.0002)	-
Correlation with the summative outcome			
Within the same topic	72.2% [kappa 0.27]	68.8% [kappa 0.22]	58.1% [kappa 0.18]
Overall outcome	73.8% [kappa 0.28]	72.2% [kappa 0.27]	48.1% [kappa 0.16]

### Predicting summative outcomes

Using the results in Table 2, presumptive summative scores and outcomes were generated and compared with the actual summative outcomes. The results are shown in the lower part of Table 2. Overall, the presumptive summative outcomes had either slight or fair agreement with the actual outcomes (Cohen’s kappa range from 0.16 – 0.28), with Medical Bacteriology having a better correlation than Medical Mycology. Interestingly, the two Medical Bacteriology quizzes had a better correlation with the overall outcomes (encompassing all CLO topics) than the Bacteriology-specific outcome. The two Medical Bacteriology quizzes tended to overestimate the number of students who passed, while the Mycology quiz tended to underestimate the number.

### Comparison between unregulated and regulated online quizzes

As the correlation between the online quiz and the summative results was poor (Table 2), adjustments were made to the Mycology quiz to implement some regulations. The comparison between the unregulated and regulated online quizzes is shown in Table 3. By asking the students to come and take the test in the designated area, the correlation between the online quiz and the summative outcomes improved drastically. Although Cohen’s kappa correlation score remained poor.

**Table 3:** Comparison between unregulated and regulated online quizzes

Parameters	Unregulated (2022)	Regulated (2023)
Attempts to pass	-	-
Time to first pass	- 0.3 (*p < *0.0001)	- 0.3 (*p < *0.0001)
First passing score	-	-
Total tries	-	-
Pass/total tries	-	-
Attempts after the first pass	10.3 (*p* = 0.0002)	-
Improvement after the first pass	-	-
Correlation with the summative outcome		
Within the same topic	58.1% [kappa 0.18]	64.8% [kappa 0.26]
Overall outcome	48.1% [kappa 0.16]	85.2% [kappa 0.23]

## Discussion

The integration of online quiz platforms into student evaluation processes has revolutionized assessment practices, offering unprecedented flexibility and alignment with competency-based learning principles [3]. However, there remains a concern that the results of the online quizzes may not be an accurate representation of the student’s true competence, as they are rather difficult to regulate. Of particular concern is that the student may solicit assistance from peers or use false credentials to take tests on their behalf [11].

Having this concern in mind, the current curriculum at the Faculty of Medicine Siriraj Hospital, Mahidol University retains the traditional, well-regulated summative examination at the end of each course to ensure the accuracy of their evaluation. However, this undermines the aim of the curriculum to become competency-based [3]. To address this challenge, this study aimed to improve the accuracy of online quizzes in evaluating the students’ knowledge competence, by including several test parameters other than the final score, to see if they can replace the traditional summative examination, thereby facilitating the curriculum’s transformation into a competency-based framework.

In this study, the first passing scores (scores of 80% or higher) of all three quizzes did not correlate with the summative result. This confirms, in part, that the quiz score is not an accurate predictor of students’ knowledge competence. However, several other parameters were found to be associated with better summative outcomes, which can be simplified into two preferable test-taking behaviors. First, the students who passed the quizzes quickly (demonstrated by the negative correlation with the number of attempts and the time duration needed before receiving a passing score) tended to do better in the summative examination. Students with this behavior were inclined to take the quizzes very early, as demonstrated by the first small peak in Fig. 1. A few of them even completed the quizzes before attending the lecture on the respective topics. Many of these students not only went on to pass the summative examination but also received an “outstanding” grade. This behavior suggests that these were students who were more academically adept.

Second, the students who improved themselves after passing the quizzes (demonstrated by the positive correlation with the presence of test attempts or the score improvement after receiving a passing score) also tended to do better in the summative examination. Many of these students required multiple attempts before receiving a passing score. Then they re-took the quizzes to receive even better scores. This behavior suggests that these were diligent students. Based on the correlation and the p values in Table 2, it is suggested that diligence also contributes to the success of Medical Bacteriology and Mycology learning. It could also be possible that these presumably diligent students, who took more attempts in the milestone quizzes, did well in the summative exam because they were more familiar with the test format, i.e. they were more test-wise, given that the test format of both assessments was similar. However this was less likely, as the total number of attempts was not associated with the increase in the summative score, but rather whether the students attempted the quizzes after passing and made some improvement.

The rationale behind the assessment of intelligence vs diligence came from previous psychological studies suggesting that the emphasis on effort rather than innate ability has more benefit for the student’s motivation and performance [12, 13]. Although this study did not assess student’s motivation nor their long-term performance beyond the current course, it emphasizes that students can also achieve learning outcomes despite their innate abilities should they invest in their efforts for improvement.

Although a few predicting factors were shown to be strongly associated with better summative scores, the calculated summative outcomes based on these factors did not correlate well with the actual outcomes (Cohen’s kappa range 0.16 – 0.28) with a great variation between different quizzes. This suggests that using online quizzes alone cannot yet replace standard summative evaluation. This study attempted to account for some concerns regarding the online quizzes by applying a regulation protocol in the subsequent Academic Year. This regulation improved the correlation but was still insufficient to provide accurate outcomes. Additional measures must be implemented to aid the evaluation of knowledge competency. Based on the programmatic assessment scheme, the employment of multiple evaluation methods, such as online quizzes together with in-class performance evaluation (class participation, class worksheet, etc.), may improve the assessment quality [14]. Further studies are needed to see if this approach could finally replace the standard summative evaluation.

Although online quizzes cannot yet substitute summative examinations, they are not without merits. First, they can help encourage students to continuously review their course materials rather than cram at the end of the course. Also, the predictive parameters in Table 2 can be used for student monitoring. A previous study reports that the student’s test-taking effort generally decreases towards the end of the examination [15]. Thus, reducing the number of test items will likely increase the accuracy of the assessment. These “smaller tests” can be used as knowledge assessment activities throughout the course to help identify students at risk, for whom interventions can be implemented early on [16]. This would greatly help both the students and the faculty members. However, the number of these smaller tests should be carefully regulated, as the higher number of tests is also correlated with test anxiety even when the stakes are low [17].

There are a few limitations in this study. First, this study uses the summative score as the sole marker of knowledge competence [1], which is not accurate in itself. The current trend now follows the programmatic assessment framework, which suggests that students’ competence should be determined based on multiple data points obtained using various methods rather than a single examination [8]. However, this study was observational and only aimed to evaluate the existing system. Second, this study used only two topics from the Medical Bacteriology and Mycology course, which, in turn, is only a single subject in the entire curriculum. Thus, it is not a representation of the medical curriculum. Still, it may provide insights regarding the evaluation of knowledge competence, especially for basic science courses with similar knowledge outcomes, such as other fields in Medical Microbiology and, to a lesser extent, other courses focusing on recall knowledge [18].

To improve upon this study, further studies can look at the implementation of other modes of assessments, such as Rubriks during group activities and peer teaching, in keeping with the programmatic assessment framework [8]. Studies can also focus on the comparison of alternative endpoints, such as the implementation of computer adaptive testing [19], to the current criterion-based scoring system. The ultimate goal of these additional studies is to further incorporate assessment into learning activities, changing the student’s view of assessment from an obstacle they have to overcome into a learning experience to help improve their competency.

## Conclusion

At the current state, the use of online quizzes alone is not sufficient for the assessment of knowledge competency. However, the identification of several testing parameters, as well as the incorporation of various assessment modalities may alleviate the need for a formal summative examination at the end of the course. Furthermore, as smaller online quizzes could be incorporated throughout the course predicting parameters obtained from these quizzes may be useful for student monitoring and early identification of students at risk, allowing for a more timely intervention.

## Data Availability

The data used in this study are available from the corresponding author upon request.

## Abbreviations

CLO: Course learning outcome
SBA-MCQ: Single-best answer multiple choice question
